# Pharmacokinetics and safety of niraparib in patients with moderate hepatic impairment

**DOI:** 10.1007/s00280-021-04329-8

**Published:** 2021-07-29

**Authors:** Mehmet Akce, Anthony El-Khoueiry, Sarina A. Piha-Paul, Emeline Bacque, Peng Pan, Zhi-Yi Zhang, Reginald Ewesuedo, Divya Gupta, Yongqiang Tang, Ashley Milton, Stefan Zajic, Patricia L. Judson, Cindy L. O’Bryant

**Affiliations:** 1 Winship Cancer Institute of Emory University, Atlanta, GA USA; 2 University of Southern California Norris Comprehensive Cancer Center, Los Angeles, CA USA; 3 University of Texas MD Anderson Cancer Center, Houston, TX USA; 4 GlaxoSmithKline, Waltham, MA USA; 5 Present Address: EQRx, Cambridge, MA USA; 6 Present Address: Dyne Therapeutics, Waltham, MA USA; 7 Present Address: Mersana Therapeutics, Cambridge, MA USA; 8 GlaxoSmithKline, Upper Providence, PA USA; 9 University of Colorado Cancer Center, Mail Stop C238, 12850 East Montview Blvd., V20-1223, Aurora, CO 80045 USA

**Keywords:** Niraparib, Pharmacokinetics, Safety, Dosing, Hepatic impairment

## Abstract

**Purpose:**

The purpose of this study is to characterize niraparib pharmacokinetics (PK) and safety in patients with normal hepatic function (NHF) versus moderate hepatic impairment (MHI).

**Methods:**

Patients with advanced solid tumors were stratified by NHF or MHI (National Cancer Institute-Organ Dysfunction Working Group criteria [bilirubin > 1.5–3 × upper limit of normal and any aspartate aminotransferase elevation]). In the PK phase, all patients received one 300 mg dose of niraparib. In the extension phase, patients with MHI received niraparib 200 mg daily; patients with NHF received 200 or 300 mg based on weight (< 77 kg, ≥ 77 kg)/platelets (< 150,000/µL, ≥ 150,000/µL). PK parameters included maximum concentration (C_max_), area under the curve to last measured concentration (AUC_last_) and extrapolated to infinity (AUC_inf_). Safety was assessed in both phases. Exposure–response (E–R) modeling was used to predict MHI effects on exposure and safety of niraparib doses ≤ 200 mg or 300/200 mg or 200/100 mg weight/platelet regimens.

**Results:**

In the PK phase (NHF, *n* = 9; MHI, *n* = 8), mean niraparib C_max_ was 7% lower in patients with MHI versus NHF. Mean exposure (AUC_last_, AUC_inf_) was increased by 45% and 56%, respectively, in patients with MHI without impacting tolerability. In the extension phase (NHF, *n* = 8; MHI, *n* = 7), the overall safety profile was consistent with previous trials. In patients with MHI, E–R modeling predicted niraparib 200 mg reduced Grade ≥ 3 thrombocytopenia incidence, whereas a 200/100 mg regimen yielded exposures below efficacy-associated levels in 15% of patients.

**Conclusion:**

These findings support adjusting the 300 mg niraparib starting dose to 200 mg QD in patients with MHI.

**Trial registration:**

NCT03359850; registered December 2, 2017

**Supplementary Information:**

The online version contains supplementary material available at 10.1007/s00280-021-04329-8.

## Introduction

Niraparib is a poly(ADP-ribose) polymerase (PARP) inhibitor approved in several countries and regions, including the USA, Canada, Japan, and EU for the first- or second-line maintenance treatment of adult patients with advanced or recurrent epithelial ovarian, fallopian tube, or primary peritoneal cancer who are in a complete or partial response to platinum-based chemotherapy [1, 2]. In the USA, niraparib is also indicated for the treatment of adult patients with advanced, homologous recombination deficiency (HRD)-positive ovarian, fallopian tube, or primary peritoneal cancer who have received ≥ 3 prior chemotherapy regimens. HRD-positive status is defined by either a deleterious or suspected deleterious *BRCA* mutation or genomic instability in patients who progressed > 6 months after response to the last platinum-based chemotherapy [1].

As of the time of this publication, niraparib is administered orally at a recommended dose for first-line maintenance treatment of 300 mg once daily (QD) for patients weighing ≥ 77 kg and with a platelet count ≥ 150,000/μL, and 200 mg QD for patients weighing < 77 kg and/or with a platelet count < 150,000/μL [1, 3]. This individualized dosing schedule reduces the risk of Grade ≥ 3 thrombocytopenia without reducing progression-free survival in patients receiving the 200 mg starting dose [3]. Niraparib 300 mg QD is indicated for the second-line maintenance treatment of advanced gynecologic malignancies, although in the EU, 200 mg may be considered for patients weighing < 58 kg [1, 2]. Moreover, niraparib 300 mg QD has been evaluated as fourth-line or later therapy in patients with metastatic, relapsed, high-grade serous epithelial ovarian, fallopian tube, or primary peritoneal cancer [4]. Niraparib has a molecular weight of 510.61 amu, an estimated logP value of 2.46, and the absolute bioavailability is approximately 73% [1, 2, 5]. Prior characterization of niraparib pharmacokinetics (PK) in patients with solid tumors demonstrated that systemic exposure increases in a dose-proportional manner and niraparib has a high volume of distribution as well as a long elimination half-life (48–51 h) [2, 6–8]. In patients with cancer, niraparib is primarily metabolized by carboxylesterases into a major metabolite (inactive), which then undergoes glucuronidation [7]. Over 21 days following administration of a single oral 300 mg dose of radiolabeled niraparib in patients with cancer who had adequate renal and hepatic function, 47.5% of the administered dose was recovered in urine and 38.8% in feces, indicating that both renal and hepatobiliary pathways are involved in the elimination of niraparib and its metabolites [7].

Hepatic impairment is common in patients with cancer and may arise from disease metastasis, advanced age, and/or treatment-related adverse effects. In patients with hepatic impairment, systemic drug level can either be increased or decreased, which may require dose adjustment to ensure optimal efficacy while avoiding unwanted toxicities [9]. As niraparib is metabolized extensively in the liver and eliminated primarily through the hepatobiliary and renal routes [7], it is important to evaluate drug PK and safety in the presence of hepatic dysfunction. In a population PK analysis (*N* = 512) of patients from a Phase 1 dose-escalation and expansion trial and the Phase 3 ENGOT-OV16/NOVA trial in patients with platinum-sensitive advanced/recurrent gynecologic malignancies, mild hepatic impairment (≤ 1.5 × upper limit of normal [ULN] total bilirubin or > ULN aspartate aminotransferase [AST] with normal bilirubin) did not impact niraparib PK [10] (manuscript in preparation). However, there are limited data available from niraparib clinical trials in patients with moderate or severe hepatic dysfunction.

The current study was undertaken to gain a better understanding of the PK profile of niraparib in patients with moderate hepatic impairment (MHI), defined by National Cancer Institute-Organ Dysfunction Working Group Criteria (NCI-ODWG) criteria (bilirubin > 1.5 × to 3 × ULN and any AST elevation). The NCI-ODWG criteria classify patients into four groups: normal hepatic function (NHF), mild, moderate, or severe hepatic impairment, based on total bilirubin and AST levels. Per these criteria, patients with moderate or severe hepatic impairment correspond to the Child–Pugh groups B and C, in whom dose modifications for chemotherapy may be required [11]. Use of NCI-ODWG criteria allows a simple and objective way to measure hepatic dysfunction in patients with cancer [11, 12].

The aim of this study was to characterize the PK and to evaluate the safety of a single dose of niraparib administered in patients with advanced solid tumors and NHF versus MHI. We also present the safety of continuous daily dosing of niraparib in these patient groups. Finally, we report the results of an independent, complementary modeling and simulation analysis undertaken to provide quantitative predictions around the potential impacts of dose adjustment on niraparib exposure and safety in patients with MHI.

## Materials and methods

### Study design and patients

This was a Phase 1, open-label, non-randomized, parallel-group, multicenter, single-dose study (NCT03359850) with optional subsequent continuous dosing. Eligible patients had advanced solid tumors that failed standard therapy or for which standard therapy was unlikely to provide meaningful benefit were aged ≥ 18 years and had an Eastern Cooperative Oncology Group (ECOG) performance status of 0 or 1. Patients with any of the following were not eligible for the study: receiving palliative radiotherapy within 1 week of study drug administration, encompassing > 20% of the bone marrow; initiation of chemotherapy within 3 weeks of study drug administration; known hypersensitivity to niraparib; receipt of colony-stimulating factors or recombinant erythropoietin within 2 weeks prior to the first dose of study treatment; symptomatic uncontrolled brain or leptomeningeal metastases; major surgery within 3 weeks of starting the study or not recovered from any effects of major surgery; considered poor medical risk due to a serious, uncontrolled medical disorder (other than hepatic impairment) or active, uncontrolled infection; received a transfusion (platelets or red blood cells) within 3 weeks of receiving niraparib; known history of myelodysplastic syndrome or acute myeloid leukemia.

Patients were stratified into groups based on hepatic function per NCI-ODWG criteria. Patients assigned to the NHF group had no history of hepatic impairment, including but not limited to chronic hepatitis C or chronic hepatitis B and had total bilirubin and AST ≤ ULN and International Normalized Ratio (INR) ≤ 1.5 × ULN unless the patient was receiving anticoagulant therapy and the INR was within therapeutic range. Patients also had adequate hematologic and renal function as defined by absolute neutrophil count (ANC) ≥ 1500/μL, platelets ≥ 100,000/μL, hemoglobin ≥ 9 g/dL and serum creatinine ≤ 1.5 × ULN or a calculated creatinine clearance ≥ 60 mL/min using the Cockcroft–Gault equation.

Patients in the MHI group had total bilirubin > 1.5 × to 3 × ULN for ≥ 2 weeks prior to Day 1 and any degree of AST elevation, INR ≤ 1.8 unless the patient was receiving anticoagulant therapy and the INR was within therapeutic range of intended use of anticoagulants, and stable MHI according to the Investigator (no clinically significant change in hepatic disease status within 30 days prior to screening). Patients in the MHI group had hematologic and renal function as defined by ANC ≥ 1000/μL, platelets ≥ 75,000/μL, hemoglobin ≥ 8 g/dL, and serum creatinine ≤ 1.5 × ULN or a calculated creatinine clearance ≥ 60 mL/min using the Cockcroft–Gault equation. Additional exclusion criteria for patients with MHI included: hepatic encephalopathy, severe portal hypertension and/or porto-systemic shunt; fluctuating or rapidly deteriorating hepatic function as determined by the investigator within the screening period; acute liver disease caused by drug toxicity or by an infection; biliary obstruction or other causes of hepatic impairment not related to parenchymal disorder and/or disease of the liver; esophageal variceal bleeding within the past 2 months; receipt of anticoagulant therapy with warfarin or related coumarins; or a history of hepatic transplant, systemic lupus erythematosus, or hepatic coma.

The study was conducted in accordance with the Declaration of Helsinki and Good Clinical Practice guidelines following approval by ethics committees and institutional review boards at each study site. All patients provided written informed consent.

### Treatment

#### PK phase

All patients received a single 300 mg dose of niraparib administered as 3 × 100 mg capsules on Day 1, following a 12-h overnight fast. Patients were permitted to resume their regular diet 4 h after niraparib administration.

#### Extension phase

Upon completion of final study assessments for the PK phase, patients were eligible to continue niraparib in the extension phase of the study if the Investigator believed this to be in the best clinical interest of the patient. Patients with NHF were dosed with niraparib based on body weight and/or baseline platelet count per the approved indication; those with a screening actual body weight of ≥ 77 kg and current platelet count of ≥ 150,000/μL at Cycle 1 Day 1 (or at screening within 72 h prior to Cycle 1 Day 1 of the extension phase) received niraparib at an oral dose of 300 mg (3 × 100 mg capsules) QD, while those with a screening actual body weight of < 77 kg and/or current platelet count of < 150,000/μL received niraparib 200 mg QD (2 × 100 mg capsules). Patients with MHI received niraparib 200 mg QD (2 × 100 mg capsules).

Treatment in the extension phase continued until disease progression (assessed by Response Evaluation Criteria in Solid Tumors [RECIST] v1.1 [13] and clinical signs and symptoms), unacceptable toxicity, death or discontinuation from the study.

### Endpoints

The primary endpoint of the study was the characterization of the niraparib PK profile after receiving a single dose of niraparib in patients with NHF compared with those with MHI. Secondary endpoints were the safety of a single dose of niraparib in patients with MHI during the PK phase, and the safety of continuously dosed niraparib in the extension phase.

### Assessments

#### Pharmacokinetics

In the PK phase, patients underwent PK sampling following niraparib administration for assessment of observed maximum plasma concentration (C_max_), area under the concentration–time curve calculated to last measured concentration (AUC_last_), area under the concentration–time curve extrapolated to infinity (AUC_inf_), time to maximum plasma concentration (t_max_), terminal half-life (*t*_½_), apparent total clearance (CL/F), apparent volume of distribution after administration (Vz/F), and terminal elimination rate constant (K_el_). Blood samples for PK evaluation were drawn pre-dose (within 30 min prior to dosing), and at 1, 2, 3, 4, 6, 8, 12, 24, 48, 72, 120, and 168 h post dose. Niraparib concentrations in these samples were determined using a validated method of liquid chromatography coupled to tandem mass spectrometry detection (LC–MS/MS) [6]. No blood samples for PK evaluation were drawn in the extension phase.

#### Safety

Safety assessments were conducted daily during the PK phase and included treatment-emergent adverse events (TEAEs), serious TEAEs and adverse events of special interest (AESIs; myelodysplastic syndromes and acute myeloid leukemia, secondary cancers, pneumonitis and embryo-fetal toxicity). During the extension phase, patients underwent safety assessments including TEAEs on Days 8, 15, and 21. Thereafter, safety was evaluated on the first day of every treatment cycle (28 ± 3 days) and at end of study. Adverse events were captured through 30 days after cessation of study treatment, and serious adverse events were captured through 90 days after cessation of study treatment (or to a minimum of 30 days post treatment if the patient started alternative anticancer therapy). During the extension phase, niraparib dose modifications could be implemented by the treating physician at any time for any grade of toxicity. Treatment with niraparib was interrupted for any treatment-related nonhematologic Common Terminology Criteria for Adverse Events (CTCAE) v4.03 Grade 3 or 4 events. If toxicity was resolved to Grade ≤ 1 within 28 days of interruption, the patient resumed treatment with niraparib at a reduced dose level. Dose interruption and modification criteria for niraparib for any hematologic toxicities were based on blood counts. Of note, the hematologic toxicity thresholds for dose interruption and reduction were different in the two groups due to patient characteristics.

### Population pharmacokinetic and exposure–response modeling

Following analysis of data from the PK phase of this study, complementary modeling and simulation analyses were conducted to further explore the potential impact of niraparib dose reductions (≤ 200 mg) on exposure and safety in patients with MHI. Specifically, population pharmacokinetic (pop-PK) and exposure–response (E–R) modeling for the safety endpoint of Grade ≥ 3 thrombocytopenia was conducted. The pop-PK model, previously developed with data from NCT00749502 [6], NOVA [14], QUADRA [4], and PRIMA [15] studies, was used to simulate niraparib exposure for virtual populations of patients with NHF and MHI (manuscript in preparation). Exposures for the virtual patients with MHI were assumed to be 56% higher than those in patients with NHF, based on results from the hepatic impairment study described herein. In the E–R analysis, the safety endpoint was the occurrence of Grade ≥ 3 thrombocytopenia and treated as a binary variable. This pop-PK analysis included data from patients who received 200 mg or 300 mg starting doses of niraparib in the PRIMA trial (*N* = 480) and used steady-state AUC (AUC_ss_) based on starting dose. The final E–R model for Grade ≥ 3 thrombocytopenia was then used to predict the probability of Grade ≥ 3 thrombocytopenia under various dosing scenarios, including reduced dosing regimens for patients with MHI. The simulated exposures in patients with MHI for reduced dosing regimens were also compared against the model-predicted exposures for patients in the PRIMA study.

### Statistical analysis

Baseline demographics and patient characteristics were summarized descriptively. PK data were analyzed using non-compartmental analysis (Phoenix WinNonlin™ v8.0; Certara, Princeton, NJ). PK parameters were summarized descriptively including the number of observations, arithmetic mean, standard deviation (SD), percent of coefficient variation (%CV), median, minimum, maximum, geometric mean, and geometric %CV. Linear models were applied to the log-transformed C_max_, AUC_last_, and AUC_inf_ with hepatic function as the independent variable. Point estimates and 90% confidence intervals (CIs) for differences between means in PK parameters on the log scale were exponentiated to express the results as ratios of geometric means, using an analysis of variance model with MHI group as fixed effect. Adverse events were listed and coded according to the current version of the Medical Dictionary for Regulatory Activities (MedDRA) and were assessed by the investigator for severity according to CTCAE v4.03. The E–R analysis dataset was assembled using SAS v9.4 (Cary, North Carolina). R v4.0.2 was used for E–R modeling and simulations and for graphical analysis, model diagnostics, and statistical summaries. Logistic regression modeling was used to investigate the E–R relationship for Grade ≥ 3 thrombocytopenia and included assessment of relevant covariates.

## Results

### Patients

A total of 17 patients were enrolled and received a single 300 mg dose of niraparib. Nine patients were assigned to the NHF group and eight patients to the MHI group, all of whom were included in the PK and safety population of the PK phase. Within the NHF group, four patients had gastrointestinal stromal tumors, two patients had prostate cancer, and one patient each had liver cancer, endometrial cancer, and pancreatic cancer. In the MHI group, three patients had liver cancer, two patients had pancreatic cancer, and one patient each had a gastrointestinal stromal tumor, adenoid cystic carcinoma, and a malignant genital neoplasm. Fifteen patients entered the extension phase of the study (NHF, *n* = 8; MHI, *n* = 7) and were assessed for safety. The data cutoff date for these analyses was April 3, 2020 (longest duration of exposure, 19 months in the extension phase). Baseline demographics and patient characteristics by group are shown in Table 1.

**Table 1:** Baseline demographics and patient characteristics by group

Characteristic	Normal hepatic function (*n* = 9)	Moderate hepatic impairment (*n* = 8)
Median age (range), years	66 (56–76)	65 (50–74)
Female, *n* (%)	2 (22)	4 (50)
Race, *n* (%)		
White	8 (89)	8 (100)
Black or African American	1 (11)	0
Mean weight (SD), kg	91.2 (19.2)	74.9 (13.3)
Tumor stage, *n* (%)		
Stage IV (NOS)	7 (78)	7 (87.5)
Stage IVB	2 (22)	1 (12.5)
Median prior lines of therapy (range)	5 (1–8)	4 (1–9)
ECOG PS, *n* (%)		
0	3 (33)	1 (12.5)
1	6 (67)	7 (87.5)
Median albumin (range), g/L	41 (35–46)	29 (22–38)
Median bilirubin (range), µmol/L	6.8 (5–14)	41.9 (29–51)
Medial ALT (range) U/L	19.0 (9–38)	50.5 (29–94)
Median AST (range) U/L	22.0 (15–46)	79.5 (47–313)
Median platelet count (range), × 10^9^/L	188.0 (143–442)	213.0 (80–252)
Median absolute neutrophil count (range), × 10^9^/L	5.1 (3–10)	5.2 (2–12)
Median hemoglobin (range), g/L	124.0 (96–141)	113.5 (89–143)

*ALT* alanine aminotransferase, *AST* aspartate aminotransferase, *ECOG* *PS* Eastern Cooperative Oncology Group performance status, *NOS* not otherwise specified

### PK phase

#### PK

**Fig. 1:**
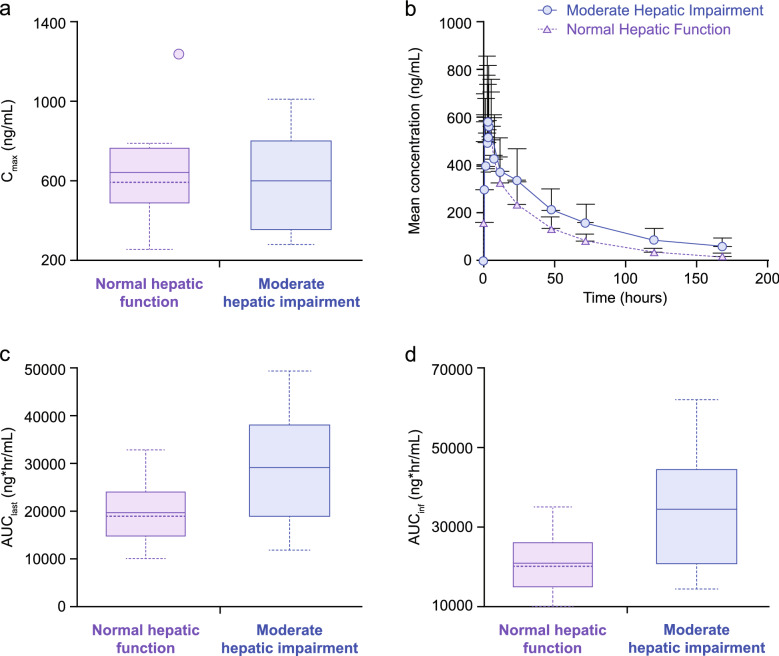
Niraparib **a** C_max_ (ng/mL) box plot, **b** mean concentration–time profiles, **c** AUC_last_ box plot and **d** AUC_inf_ box plot following single-dose administration by hepatic function group. **a**, **c,** and **d** solid lines represent mean values; dashed lines represent median values; upper and lower boxes represent middle two quartiles (i.e., 50%); whiskers indicate minimum and maximum values. **a** Circle indicates outlier value in normal hepatic function group. **b** Error bars indicate ± SD. *AUC*_*inf*_, area under the plasma concentration–time curve from time 0 extrapolated to infinity; *AUC*_*last*_ area under the plasma concentration–time curve from time 0 to the time of the last quantifiable concentration; *C*_*max*_ observed maximum plasma concentration; *SD* standard deviation

A detailed summary of the PK parameters of niraparib by hepatic function is given in Supplementary Table S1. Mean C_max_ was 644 ng/mL for patients with NHF and 601 ng/mL for patients with MHI (Fig. 1a). Mean (SD) niraparib concentrations over time by hepatic function group are shown in Fig. 1b; an increase in niraparib exposure was observed in patients with MHI versus those with NHF.

Maximum plasma concentrations of niraparib were achieved by approximately 4 h (median t_max_) for both groups. Overall exposure, in terms of AUC_inf_, was 20,900 ng × h/mL for patients with NHF and 34,300 ng × h/mL for patients with MHI. Both CL/F and Vz/F were higher in patients with NHF than in those with MHI. Patients with MHI had a longer niraparib *t*_1/2_ of 56.2 h, compared with 44.1 h for patients with NHF. Intersubject variability in the PK parameters, as measured by %CV, ranged from 11.2 to 42.8% for patients with NHF and 24.1 to 50.8% for patients with MHI.

Box plots of niraparib PK parameters (AUC_last_ and AUC_inf_) are presented in Fig. 1c, d, and Table 2 provides a statistical comparison of C_max_, AUC_last_, and AUC_inf_ in patients with MHI versus those with NHF. There was a 7% reduction in C_max_ in patients with MHI versus those with NHF; these results suggest that MHI had minimal impact on niraparib C_max_. On average, niraparib AUC_last_ and AUC_inf_ were increased by 45% and 56%, respectively, in patients with MHI compared with patients with NHF, showing that a reduction in hepatic function led to greater overall exposure to niraparib.

**Table 2:** Summary of the effect of moderate hepatic impairment on niraparib PK: ratio of moderate hepatic impairment to normal hepatic function

Parameter	*n*	Mean (SE)	Geometric LS mean (SE)^a^	Ratio (moderate/normal)	
				Geometric LS mean ratio (SE)^a^	90% CI^a^
C_max_ (ng/mL)					
Moderate hepatic impairment	8	601.00 (88.96)	552.68 (86.35)	0.9305 (0.1998)	0.6386–1.3558
Normal hepatic function	9	644.00 (91.98)	593.96 (87.49)		
AUC_last_ (h × ng/mL)					
Moderate hepatic impairment	8	29,317.51 (4393.22)	26,825.19 (3948.56)	1.4528 (0.2939)	1.0190–2.0712
Normal hepatic function	9	19,545.11 (2291.79)	18,464.93 (2562.52)		
AUC_inf_ (h × ng/mL)					
Moderate hepatic impairment	7	34,265.92 (6296.04)	30,802.12 (5078.95)	1.5639 (0.3438)	1.0618–2.3034
Normal hepatic function	9	20,915.19 (2525.24)	19,696.31 (2864.21)		

*AUC*_*inf*_ area under the concentration–time curve extrapolated to infinity, *AUC*_*last*_ area under the concentration–time curve calculated to last measured concentration, *CI* confidence interval, *C*_*max*_ observed maximum plasma concentration, *SE* standard error, *LS* least squares

^a^From an ANOVA model for the log-transformed parameter results with fixed effect hepatic impairment group

#### Safety

Safety data during the PK phase were consistent with the known safety profile for niraparib (Table 3). At least one any-grade TEAE was experienced by five of nine patients in the NHF group and three of eight patients in the MHI group. The most common TEAEs of any grade (affecting ≥ 2 patients overall) were nausea, increased ALT, increased AST, and hyperbilirubinemia. Three patients with NHF had any-grade drug-related TEAEs (including abdominal distension [*n* = 2], nausea, vomiting, taste disorder, ALT increased, AST increased, amylase increased, and lipase increased [*n* = 1 each]). There were no drug-related TEAEs in the MHI group.

**Table 3:** Summary of TEAEs during the PK phase after administration of a single 300 mg dose of niraparib

TEAE^a^, *n*	Normal hepatic function (*n* = 9)	Moderate hepatic impairment (*n* = 8)
Any grade TEAE	5	3
Nausea	1	1
ALT increased	1	1
AST increased	1	1
Hyperbilirubinemia	0	2
Abdominal distension	1	0
Increased amylase	1	0
Increased lipase	1	0
Vomiting	1	0
Taste disorder	1	0
Acute respiratory failure	1	0
Pulmonary hypertension	1	0
Influenza	1	0
Pneumonia	1	0
Back pain	1	0
Peripheral neuropathy	0	1
Fatigue	0	1
Decreased appetite	0	1
Hyponatremia	0	1
Lymphopenia	0	1
Any grade drug-related TEAE	3	0
Abdominal distension	2	0
Nausea	1	0
Vomiting	1	0
ALT increased	1	0
Amylase increased	1	0
AST increased	1	0
Lipase increased	1	0
Taste disorder	1	0
Any drug-related Grade ≥ 3 TEAE	1	0
AST increased	1	0
Amylase increased	1	0
Lipase increased	1	0

*ALT* alanine aminotransferase, *AST* aspartate aminotransferase, *TEAE* treatment-emergent adverse event

^a^Patients may have had more than 1 TEAE by preferred term

Grade ≥ 3 TEAEs were reported in two of nine patients in the NHF group and one of eight patients in the MHI group. Drug-related Grade ≥ 3 TEAEs are shown in Table 3. One patient in the NHF group experienced serious unrelated TEAEs (pneumonia, influenza, pulmonary hypertension, and acute respiratory failure) and did not continue into the extension phase. One patient with NHF experienced niraparib-related TEAEs (ALT increased, AST increased, amylase increased, and lipase increased), resulting in a delay of the first extension phase dose of niraparib (recorded as a dose interruption). The patient was able to proceed with daily dosing of niraparib in the extension phase upon resolution of these TEAEs. There were no drug-related serious TEAEs during the PK phase. No TEAEs led to death, and there were no AESIs in either group during the PK phase.

### Extension phase

#### Duration of treatment exposure

Upon completion of final study assessments for the PK phase, 15 patients entered the extension phase. In the NHF group, seven patients received niraparib 300 mg QD; one patient received niraparib 200 mg QD (based on an actual screening body weight of < 77 kg and/or current platelet count of < 150,000/μL). All patients in the MHI group received 200 mg QD per protocol (regardless of screening body weight or platelet count). The median duration of exposure to niraparib was 56.5 days (range, 21–578) in the NHF group and 27.0 days (range, 6–84) in the MHI group. The median number of cycles of niraparib treatment was 2.0 (range, 1–20) and 1.0 (range, 1–3), respectively.

#### Safety

Safety data for niraparib in the extension phase are summarized in Table 4. All patients experienced at least one any-grade TEAE. Any grade drug-related TEAEs were experienced by seven of eight patients with NHF and five of seven patients with MHI. Grade ≥ 3 TEAEs were reported in four of eight patients in the NHF group and seven of seven patients in the MHI group. Drug-related Grade ≥ 3 TEAEs are also listed in Table 4. Two patients in each group had serious TEAEs (NHF: pyrexia, musculoskeletal pain [*n* = 1 each]; MHI: abdominal pain, sepsis [*n* = 1 each]). A niraparib-related serious TEAE was reported by one of eight patients in the NHF group (pyrexia).

**Table 4:** Summary of TEAEs during the extension phase (continuous daily administration of niraparib^a^)

TEAE^b^, *n*	Normal hepatic function (*n* = 8)	Moderate hepatic impairment (*n* = 7)
Any grade TEAE^c^	8	7
Fatigue	7	5
Anemia	3	5
Constipation	4	3
Thrombocytopenia	2	5
Nausea	4	1
Abdominal pain	2	3
Decreased appetite	2	3
Blood creatinine increased	2	1
Insomnia	3	0
Vomiting	1	2
Back pain	2	0
Musculoskeletal pain	2	0
Myalgia	2	0
Dizziness	2	0
Headache	2	0
Peripheral edema	0	2
Hyperbilirubinemia	0	2
Jaundice	0	2
AST increased	0	2
Hypokalemia	0	2
Any grade drug-related TEAE	7	5
Fatigue	6	2
Thrombocytopenia	2	5
Anemia	3	2
Nausea	3	1
Abdominal pain	1	0
Decreased appetite	2	1
Dizziness	2	0
Any drug-related Grade ≥ 3 TEAE	3	5
Thrombocytopenia	2	4
Anemia	2	2
Asthenia	0	1
Blood bilirubin increased	0	1
Platelet count decreased	0	1
Nausea	0	1
Any drug-related serious TEAE	1	0
Pyrexia	1	0

*ALT* alanine aminotransferase, *AST* aspartate aminotransferase, *TEAE* treatment-emergent adverse event

^a^Patients with normal hepatic function and a screening actual body weight of ≥ 77 kg and current platelet count of ≥ 150,000/μL at Cycle 1 Day 1 (or at screening within 72 h prior to Cycle 1 Day 1 of the extension phase) received niraparib 300 mg QD. Patients with normal hepatic function and a screening actual body weight of < 77 kg and/or current platelet count of < 150,000/μL received niraparib 200 mg QD. Patients with moderate hepatic impairment received niraparib 200 mg QD

^b^Patients may have had more than 1 TEAE by preferred term

^c^Occurring in ≥ 2 patients in either group

TEAEs led to niraparib dose interruption in five of eight patients with NHF (thrombocytopenia and anemia [*n* = 2 each], nausea, platelet count decreased, dizziness [*n* = 1 each]) and three of seven patients with MHI (thrombocytopenia [*n* = 2], anemia, nausea, constipation, asthenia, fatigue, peripheral edema, sepsis, platelet count decreased, blood creatinine increased, hemoptysis, pulmonary embolism [*n* = 1 each]). Niraparib dose reduction (by 100 mg QD) was reported for three of eight patients with NHF (for anemia [*n* = 2], thrombocytopenia, and fatigue [*n* = 1 each]) and one of seven patients in the MHI group (nausea).

One patient with MHI had TEAEs of abdominal pain and upper respiratory tract infection that led to treatment discontinuation. No TEAEs led to death and no AESIs were reported during the extension phase in either group.

### Exposure–response modeling

Based on the observed increased exposure of niraparib in patients with MHI in this study, further modeling and simulation analyses were performed to explore the effects of reduced niraparib doses (≤ 200 mg) on exposure (AUC_ss_) and safety, using the safety endpoint of Grade ≥ 3 thrombocytopenia. Grade ≥ 3 thrombocytopenia was selected as the safety endpoint because it was the most common hematologic event during the first 3 months of niraparib treatment in patients starting with the 300 mg dose in ENGOT-OV16/NOVA [3]. Univariate logistic regression plots were used to explore the relationships of niraparib exposure (AUC_ss_), baseline platelets, weight, and age, with the probability of Grade ≥ 3 thrombocytopenia based on data from the PRIMA study, as shown in Supplementary Figure S1. These plots indicated that the probability of Grade ≥ 3 thrombocytopenia increased with increasing niraparib exposure and age and decreased with increasing platelet counts and weight.

To develop the E–R model for Grade ≥ 3 thrombocytopenia, a full model was estimated with the following explanatory variables: AUC_ss_, baseline platelet count, baseline weight, age (treated as continuous variables); NCI-ODWG hepatic impairment category and ECOG status (categorical variables). Gender was not included as all patients in the analysis were female; race was not included due to a limited number of non-white patients. Backward deletion was performed using the stepAIC function, which applied an increase in Akaike information criterion (AIC) of any amount as criterion for deletion. After backward deletion, covariates with *P* < 0.05 were dropped from the model; as such, age, NCI-ODWG category, and ECOG status dropped out of the model. The model arising from this step was considered the final E–R model and included AUC_ss_, baseline platelets, and weight as explanatory variables.

Parameter estimates and odds ratios for the final model are presented in Supplementary Table S2. The coefficients for AUC_ss_, platelets, and weight were statistically significant; all *P *values associated with the estimated coefficients were ≤ 0.0204 and the log likelihood ratio test *P* values were ≤ 0.0175 when each coefficient was dropped from the model one at a time. This model identified that higher AUC_ss_, lower baseline body weight, and lower baseline platelet count were associated with higher probability of Grade ≥ 3 thrombocytopenia.

Simulations were performed in a virtual population of 5,000 patients with baseline characteristics similar to patients enrolled in the PRIMA study (including patients with NHF and MHI) and based on the final E–R model. For the virtual population of patients with MHI, the niraparib AUC_ss_ was multiplied by 1.56, based on the 56% increase in AUC_inf_ observed in the PK phase of this study. To assess the impact of 100 mg QD, 200 mg QD, or weight- and platelet-based dosing regimens on exposure and safety in patients with MHI, simulations were run using the virtual population for each of the following niraparib weight- and platelet-based dosing regimens: 300/200 mg (starting dose of 300 mg if weight ≥ 77 kg and platelets ≥ 150,000/µL, and 200 mg if weight < 77 kg and/or platelets < 150,000/µL); and 200/100 mg (starting dose of 200 mg if weight ≥ 77 kg and platelets ≥ 150,000/µL and 100 mg if weight < 77 kg or platelets < 150,000/µL).

**Fig. 2:**
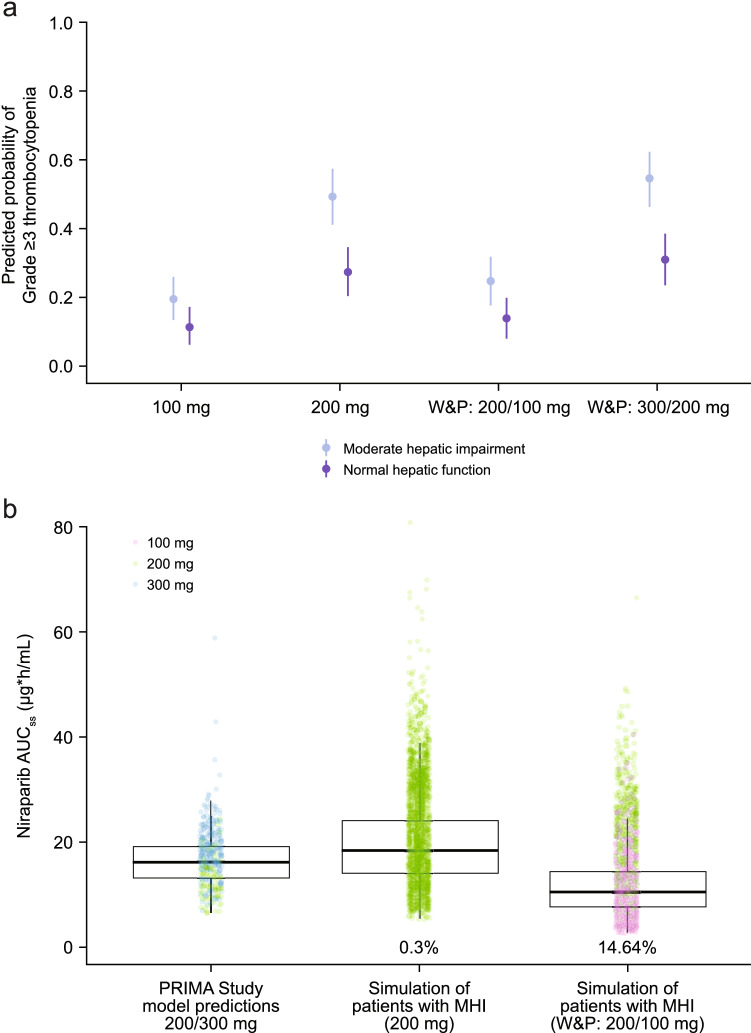
**a** Comparison of predicted probabilities of Grade ≥ 3 thrombocytopenia for patients with NHF and MHI and **b** simulated AUC_ss_ in patients with MHI and model-predicted AUC_ss_ in patients from PRIMA study. **a** Closed circles represent the mean of probability of Grade ≥ 3 thrombocytopenia and error bars represent the 95% prediction intervals. **b** Percentages represent the portion of virtual patients with AUC_ss_ below the range of AUC_ss_ in the PRIMA study. AUC_ss_ was based on starting dose. *AUC*_*ss*_ steady-state area under concentration–time curve, *MHI* moderate hepatic impairment, *NHF* normal hepatic function, W&P: X/Y mg = X mg if baseline body weight ≥ 77 kg and baseline platelet count ≥ 150,000/µL and Y mg if baseline body weight < 77 kg or baseline platelet count < 150,000/µL

In the MHI population, administering niraparib 200 mg QD to all patients or using the 300/200 mg schedule was predicted to result in a higher probability of Grade ≥ 3 thrombocytopenia (49% and 55%, respectively), compared to 300/200 mg dosing in patients with NHF (31%) (Fig. 2a). Reducing the administered dose in the MHI population to 100 mg for all patients or using 200/100 mg dosing was associated with predicted Grade ≥ 3 thrombocytopenia probabilities of 20% and 25%, respectively.

To explore the impact of 200 mg and 200/100 mg dosing regimens on efficacy in patients with impaired hepatic function, simulated AUC_ss_ for patients with MHI receiving these regimens were compared numerically and graphically using boxplots to model-predicted AUC_ss_ in patients with NHF who received 200 mg and 300 mg niraparib in the PRIMA trial (Fig. 2b). The simulated exposures in patients with MHI and the model-predicted exposures in patients from the PRIMA study overlapped. However, AUC_ss_ in patients with MHI fell below the range of model-predicted exposures in the PRIMA study in 14.6% of simulated patients (nearly all in the low weight and/or low platelet group) following 200/100 mg dosing and only in 0.3% of simulated patients following 200 mg niraparib.

## Discussion

As niraparib is metabolized extensively in the liver and eliminated via hepatobiliary and renal routes, it is important to assess PK and safety in patients with hepatic impairment. The results from the PK phase of this Phase 1 study, in which all patients received a single 300 mg dose of niraparib, demonstrated that MHI did not meaningfully impact niraparib C_max_ but resulted in an approximately 50% increase in overall exposure compared with patients with NHF. The increased exposure of niraparib following a single oral dose of 300 mg in patients with MHI did not alter the overall tolerability of niraparib in the PK phase compared with patients with NHF. Overall, the pattern of TEAEs observed in this study with niraparib is consistent with data from previous Phase 3 registrational trials (NOVA, PRIMA, and QUADRA) [4, 14–16], regardless of hepatic function.

Although the overall tolerability profile of niraparib was similar in both hepatic function groups, a higher proportion of patients in the MHI group experienced any-cause Grade ≥ 3 TEAEs than in the NHF group (*n* = 7/7 [100%] vs *n* = 4/8 [50%]) during the extension phase. However, as the MHI group had liver dysfunction (though stable) at enrollment, cases of Grade ≥ 3 AST or ALT elevations or hyperbilirubinemia might be anticipated. Indeed, hyperbilirubinemia was reported in two patients in the MHI group during the PK and extension phases, whereas no cases were reported in NHF group. More patients with MHI experienced Grade ≥ 3 thrombocytopenia or anemia. Less stringent inclusion criteria for hematologic function at study entry were applied to the MHI group (i.e., lower platelet, neutrophil, and hemoglobin counts were acceptable on entry into the study) leading to less favorable baseline blood counts compared with the normal hepatic function group (Table 1). Also, there was a lower threshold for niraparib treatment modifications related to hematologic toxicity between groups: platelets < 50,000/µL, neutrophils < 750/µL, and hemoglobin < 7 g/dL in the MHI group; < 100,000/µL, < 1000/µL, and < 8 g/dL, in the NHF group. Nearly half of patients in the extension phase (*n* = 5 NHF; *n* = 3 MHI) required dose interruptions for thrombocytopenia/platelet count decreased or anemia; however, this led to dose reductions in only three patients in the NHF group. This may also be explained by the shorter duration of niraparib treatment in the MHI group compared with the NHF group in the extension phase (range, 6–84 days; median 1 cycle vs range, 21–578 days; median 2 cycles).

Given that niraparib exposure was increased by approximately 50% in patients with MHI in the PK phase of this study, there is a clear rationale for reducing the starting dose from 300 to 200 mg QD for patients with MHI and body weight ≥ 77 kg and platelets ≥ 150,000/µL. As niraparib PK is linear in this dose range, patients with MHI receiving 200 mg niraparib have similar exposures as patients with NHF receiving 300 mg QD. The situation is more complex for low weight/low platelet patients with MHI; if the dose is not reduced, higher exposures due to hepatic impairment may result in increased thrombocytopenia. On the other hand, dose reduction may decrease efficacy. To quantitatively explore this question, pop-PK and E–R modeling were used to predict the effects of MHI on exposure and safety of niraparib ≤ 200 mg and demonstrated that there is an elevated thrombocytopenia risk due to increased niraparib exposures in patients with MHI. Thus, lowering the dose would reduce exposures and decrease the risk of thrombocytopenia. However, for approximately 15% of the simulated patients with MHI based on pop-PK exposure estimates, reducing the dose below 200 mg would also result in decreased niraparib exposures below those associated with efficacy in the PRIMA study. Based on the data available, the 200 mg dose appears to offer a reasonable option that balances exposure and anticancer efficacy with the risk of thrombocytopenia, which can be monitored and managed clinically.

In conclusion, due to increased exposure of niraparib in patients with MHI, the PK and safety data support a flat starting daily dose of 200 mg for this patient population. This starting dose should provide a similar level of niraparib exposure to the approved 300 mg QD starting dose in the NHF population. The absence of new safety signals, the low incidence of dose reductions, interruptions, or discontinuations, and the absence of deaths due to TEAEs in the extension phase for patients with MHI additionally support the 200 mg QD dosing schedule, which provides a treatment option for patients with liver dysfunction that was not previously available. Assessment of the PK and safety of niraparib in patients with severe hepatic impairment was beyond the scope of this study and requires further investigation.

## Supplementary Information

Below is the link to the electronic supplementary material.

Supplementary file1 (DOCX 821 KB)

## Data Availability

GlaxoSmithKline (GSK) makes available anonymized individual participant data and associated documents from interventional clinical studies that evaluate medicines, upon approval of proposals submitted to www.clinicalstudydatarequest.com.
