# Supplementary material for: Pharmacokinetics and safety of niraparib in patients with moderate hepatic impairment
Source: Cancer Chemother Pharmacol. 2021 Jul 29;88(5):825–36. doi: 10.1007/s00280-021-04329-8 (PMC8484145; doi:10.1007/s00280-021-04329-8)
Supplement: Supplementary file 1 — Supplementary file1 (DOCX 821 KB) [file 280_2021_4329_MOESM1_ESM.docx]

**Pharmacokinetics and Safety of Niraparib in Patients with Moderate Hepatic Impairment**

Mehmet Akce^1^, Anthony El-Khoueiry^2^, Sarina A. Piha-Paul^3^, Emeline Bacque^4^, Peng Pan^4^, Zhi-Yi Zhang^4^, Reginald Ewesuedo^4^, Divya Gupta^4^, Yongqiang Tang^4^, Ashley Milton^4*^, Stefan Zajic^5^, Patricia L. Judson^4^, Cindy L. O’Bryant^6^

^1^Winship Cancer Institute of Emory University, Atlanta, GA, USA; ^2^University of Southern California Norris Comprehensive Cancer Center, Los Angeles, CA, USA; ^3^University of Texas MD Anderson Cancer Center, Houston, TX, USA; ^4^GlaxoSmithKline, Waltham, MA, USA; ^5^GlaxoSmithKline, Upper Providence, PA, USA; ^6^University of Colorado Cancer Center, Aurora, CO, USA

*Current affiliation: Dyne Therapeutics, Waltham, MA, USA

^†^Current affiliation: Mersana Therapeutics, Cambridge, MA, USA

**Corresponding author:** Cindy L. O’Bryant

**Address:** University of Colorado Cancer Center, Mail Stop C238, 12850 East Montview Blvd., V20-1223 Aurora, CO 80045, USA

**Phone number:** [303-724-2625](tel:303-724-2625)

**Email:** cindy.obryant@cuanschutz.edu

**SUPPLEMENT**

**Supplementary Data**

**Supplementary Table S1.** Summary of PK parameters of niraparib by hepatic function group

| Group |  | C_max_  (ng/mL) | AUC_last_  (h*ng/mL) | AUC_inf_  (h*ng/mL) | t_max_ (h) | t_1/2_ (h) | CL/F (L/h) | Vz/F (L) | K_el_ (1/h) |
| --- | --- | --- | --- | --- | --- | --- | --- | --- | --- |
| Moderate hepatic impairment | n | 8 | 8 | 7 | 8 | 7 | 7 | 7 | 7 |
|  | Mean (SD) | 601 (252) | 29,300 (12,400) | 34,300 (16,700) | 4.53 (2.01) | 56.2 (13.5) | 10.9 (5.53) | 847 (428) | 0.0130  (0.00342) |
|  | Median (range) | 600  (280–1010) | 29,400 (11,800–49,900) | 31,900  (14,700  –62,100) | 4.09  (2.00–8.02) | 55.0  (36.8–69.6) | 9.39  (4.83–20.4) | 660  (484–1620) | 0.0126  (0.00996–  0.0189) |
|  | %CV | 41.9 | 42.4 | 48.6 | 44.3 | 24.1 | 50.8 | 50.5 | 26.3 |
|  | Geometric mean | 553 | 26,800 | 30,800 | 4.14 | 54.8 | 9.74 | 769 | 0.0127 |
|  | %CV Geometric mean | 47.4 | 49.6 | 54.3 | 48.2 | 25.8 | 54.3 | 48.3 | 25.8 |
| Normal hepatic function | n | 9 | 9 | 9 | 9 | 9 | 9 | 9 | 9 |
|  | Mean (SD) | 644  (276) | 19,500  (6880) | 20,900  (7580) | 4.36  (1.36) | 44.1  (5.19) | 16.2  (6.34) | 1010  (349) | 0.0159  (0.00178) |
|  | Median (range) | 594  (256–1240) | 18,900  (9520–32,900) | 20,000  (10,200–  35,300) | 4.00  (3.00–6.33) | 43.2  (37.1–53.8) | 15.0  (8.50–29.4) | 917  (618–1830) | 0.0161  (0.0129–0.0187) |
|  | %CV | 42.8 | 35.2 | 36.2 | 31.1 | 11.8 | 39.1 | 34.6 | 11.2 |
|  | Geometric mean | 594 | 18,500 | 19,700 | 4.18 | 43.9 | 15.2 | 964 | 0.0158 |
|  | %CV Geometric mean | 45.6 | \| 37.7 \|  \| \| --- \| --- \| | 38.7 | 31.3 | 11.5 | 38.7 | 31.2 | 11.5 |

AUC_last_, area under the concentration–time curve calculated to last measured concentration; AUC_inf_, area under the concentration–time curve extrapolated to infinity; CL/F, total clearance; C_max_, observed maximum plasma concentration; CV, coefficient of variation; Kel, terminal elimination rate constant; SD, standard deviation; t_½_, terminal half-life; t_max_, time to maximum plasma concentration; Vz/F, apparent volume of distribution during the terminal elimination phase.

**Supplementary Table S2.** Grade ≥3 thrombocytopenia model parameter estimates

| Parameter | Estimate | RSE% | *P-*value | Odds ratio (95% CI) |
| --- | --- | --- | --- | --- |
| Intercept | -1.110 | 59.1 | 0.0903 | – |
| AUC_ss_, µg*h/mL | 0.164 | 14.6 | <0.0001 | 1.18 (1.13–1.24) |
| Baseline platelet count, (x10^9^/L) | -0.00314 | 43.1 | 0.0204 | 0.997 (0.994–0.999) |
| Baseline body weight, kg | -0.0196 | 36.9 | 0.0068 | 0.981 (0.967–0.994) |

AUC_ss_, steady-state area under the concentration–time curve (based on starting dose); CI, confidence interval; RSE, relative standard error.

The final model equation was as follows: log(p/(1-p)) = -1.110 + 0.164 × AUC_ss_ – 0.0196 × weight – 0.00314 × platelets

where AUC_ss_ is in μg*h/mL, weight is in kg, and platelet counts are in ×10^9^/L.

**Supplementary Figure S1.** Relationship between a) AUC_ss_, b) platelets, c) weight, and d) age, and the probability of Grade ≥3 thrombocytopenia in the PRIMA study

**a)**


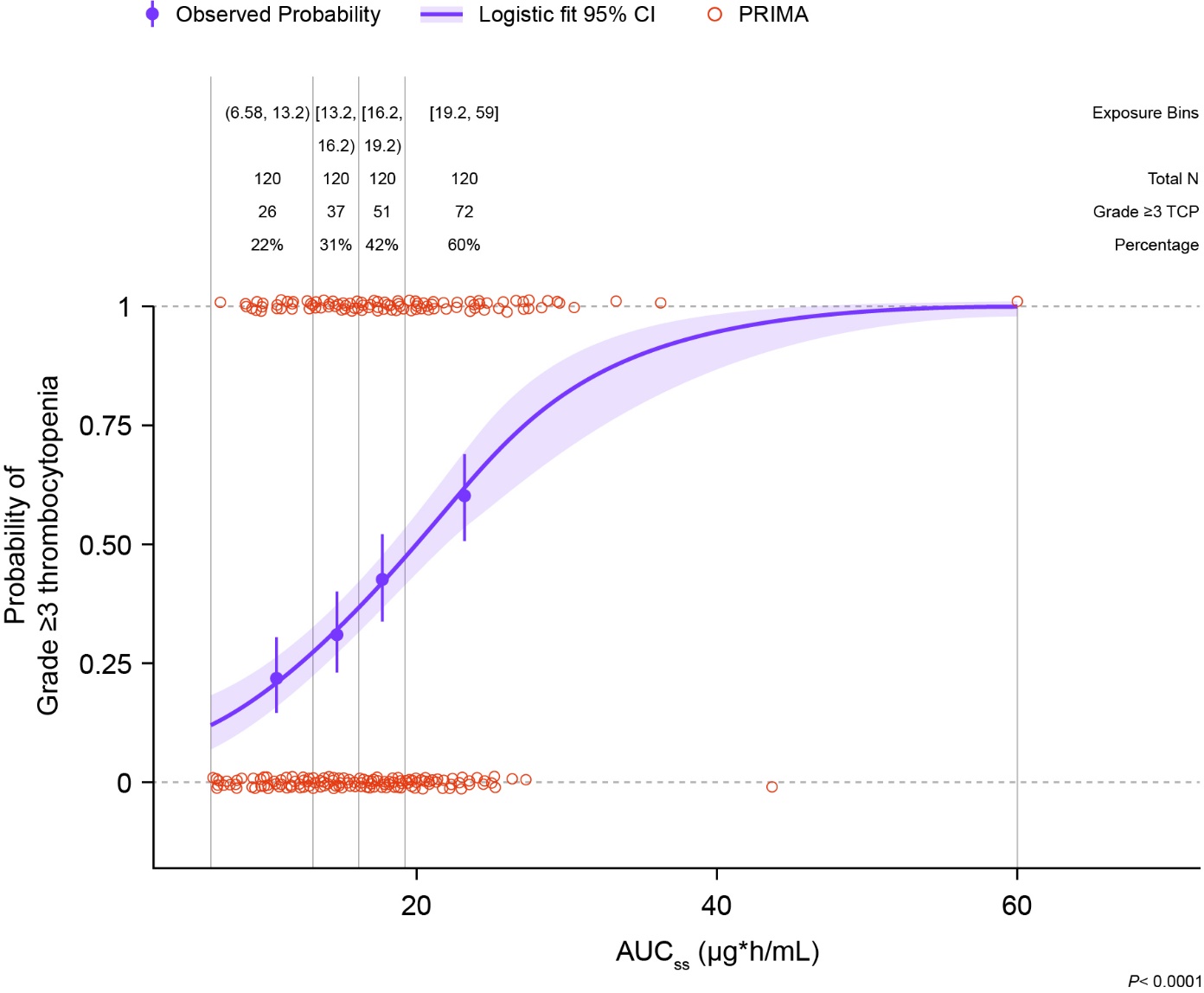


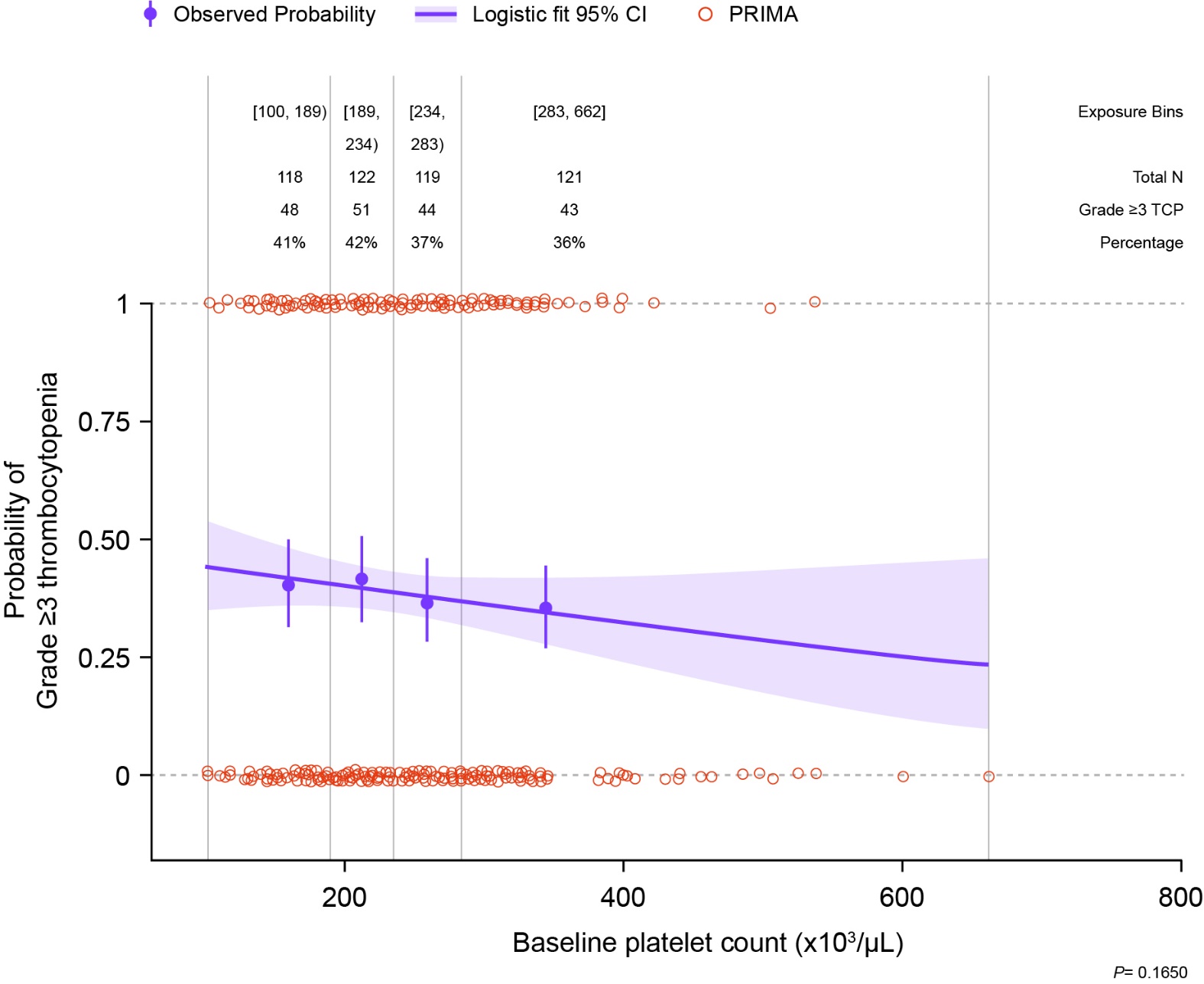
**b)**

**c)**

**
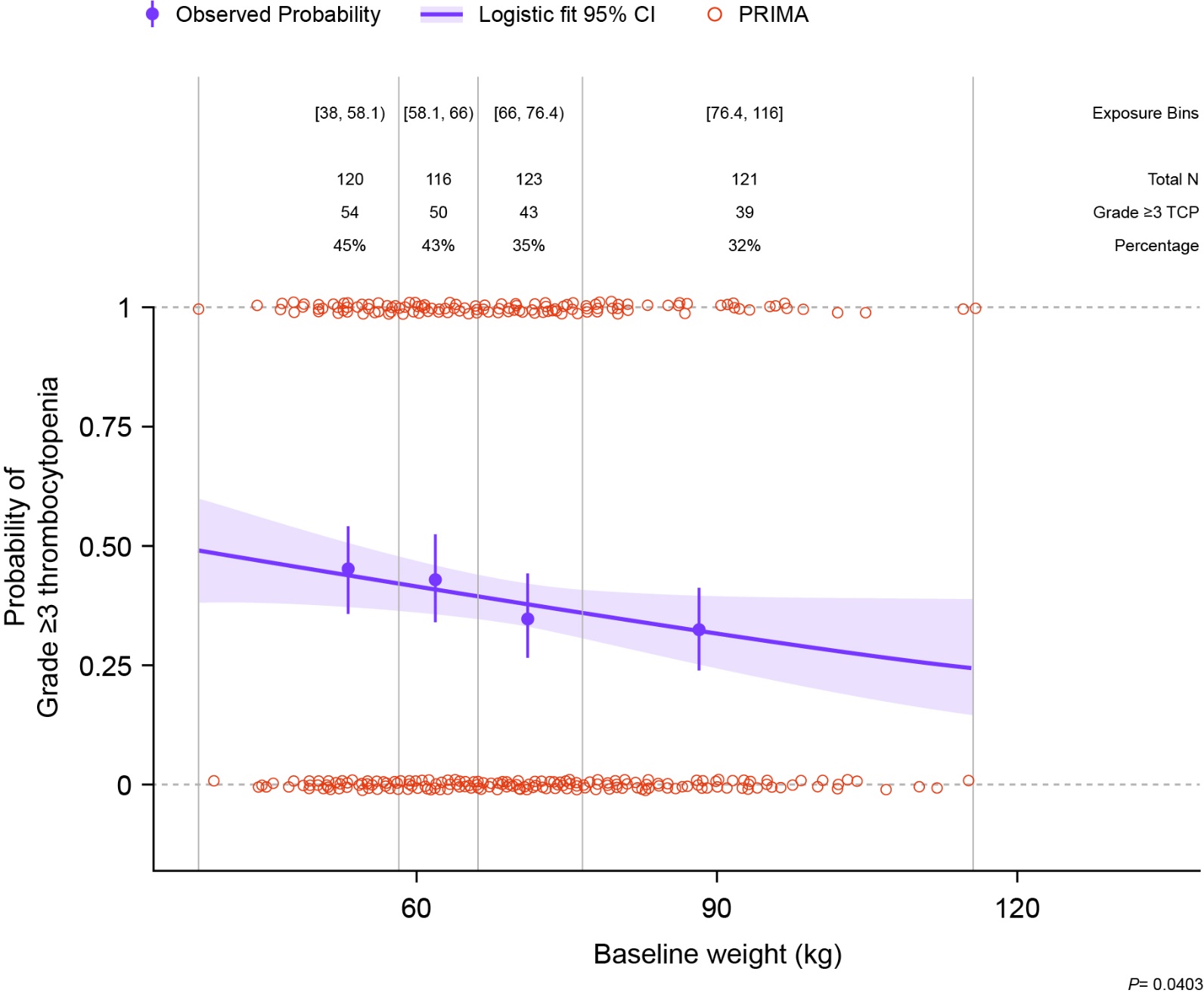
**

**d)**

**
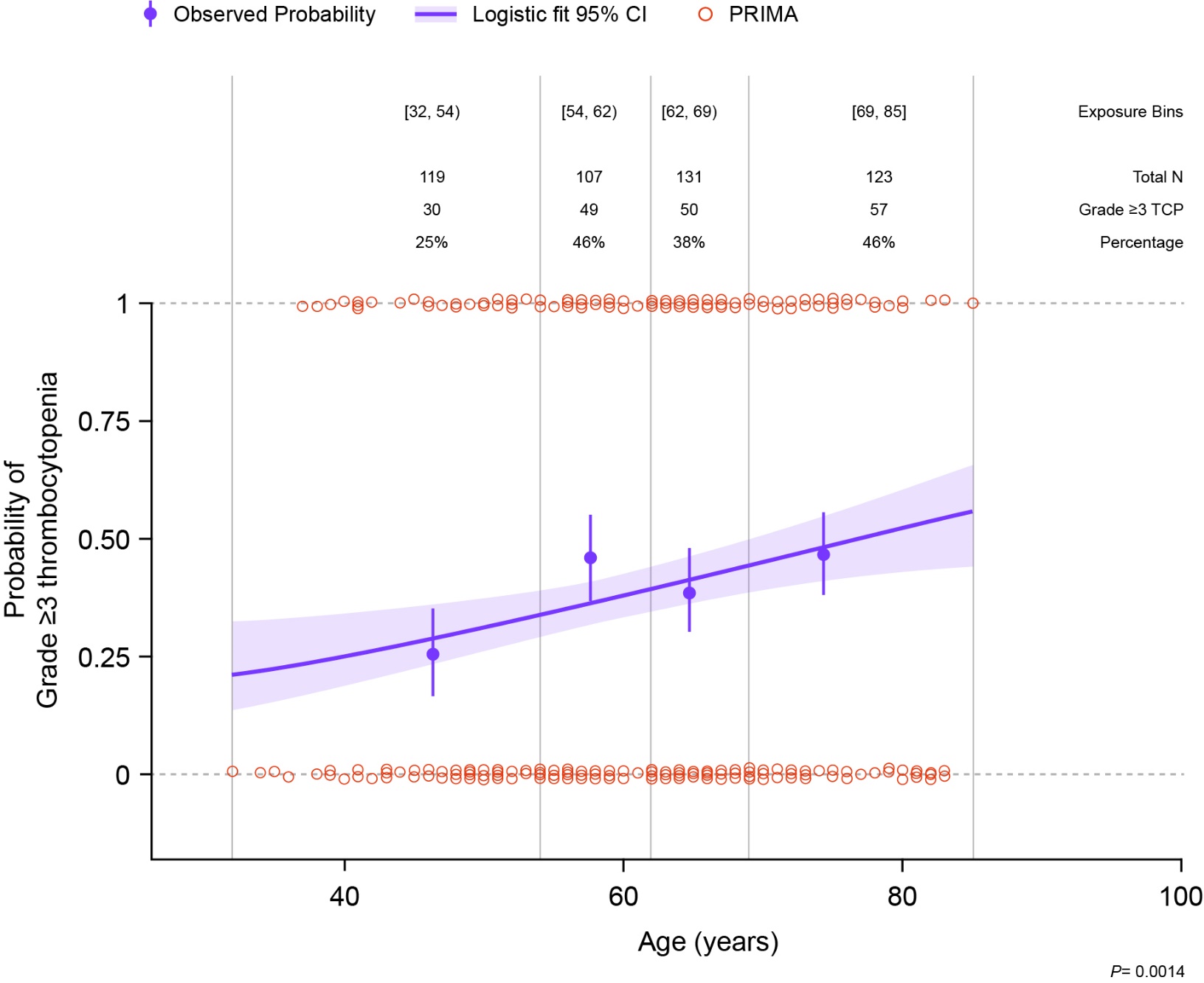
**

The independent variables were divided into 4 equally sized rank-ordered groups. Black points and error bars represent the observed proportions and 95% CIs for each exposure group (plotted at the mean exposure within each exposure group), respectively. The black curve represents the prediction of the univariate logistic regression model, and the gray shaded region represents the 95% CI of the prediction.

AUC_ss_, steady-state area under the concentration–time curve; CI, confidence interval; TCP, thrombocytopenia.
